# Evaluation of inflammatory status and macrophage polarization in premature ovarian insufficiency mice induced by chemotherapy

**DOI:** 10.3389/fendo.2026.1798400

**Published:** 2026-10-12

**Authors:** Maoxing Tang, Manzhi Zhao, Yangcheng Yao, Xiqian Zhang, Fenghua Liu

**Affiliations:** 1 Department of Reproductive Medicine, Guangdong Women and Children Hospital, Guangzhou, China; 2 Department of Reproductive Medicine, Guangdong Provincial People’s Hospital (Guangdong Academy of Medical Sciences), Southern Medical University, Guangzhou, China; 3 Department of Pulmonary and Critical Care Medicine, Guangdong Provincial People’s Hospital (Guangdong Academy of Medical Sciences), Southern Medical University, Guangzhou, China

**Keywords:** chemotherapy, inflammation, macrophage polarization, macrophages, ovarian reserve, premature ovarian insufficiency

## Abstract

**Background:**

With an increasing trend of cancer occurring in young women, chemotherapy, a commonly used anticancer method, often leads to premature ovarian insufficiency (POI) and infertility. Until now, there has been, however, no available treatment to cure POI patients caused by chemotherapy. This highlights the urgent need to unravel the mechanism underlying chemotherapy-induced POI development.

**Methods:**

The POI mouse model was established with cyclophosphamide and busulfan. The levels of key pro-inflammatory and anti-inflammatory cytokines in ovaries were determined using qPCR or immunohistochemistry (IHC), while the levels of other pro-inflammatory cytokines in the serum were measured by ELISA. The macrophages (MΦs) populations and polarization within ovaries were analyzed using flow cytometry and immunofluorescence.

**Results:**

Initially, chemotherapy-treated mice exhibited ovarian dysfunction and a decline in ovarian reserve, characterized by disrupted hormone levels and follicular loss. Following chemotherapy, there was an increase of pro-inflammatory cytokines, including *Il1b*, *Il6* and *Tnfa* in mouse ovaries. Similarly, a significant elevation of pro-inflammatory cytokines like IL-6 and IL-18 was observed in the serum of chemotherapy-induced POI mice. Moreover, we found that the proportion of CD11c^+^ M1-like MΦs was remarkably increased in ovaries of chemotherapy-induced POI mice compared to the controls. However, the proportion of CD206^+^ M2-like MΦs did not differ significantly between POI and control groups. Notably, ovarian MΦs exhibited differential distribution after chemotherapy. CD11c^+^ M1-like MΦs mainly localized around atretic follicles, while CD206^+^ M2-like MΦs predominantly accumulated in ovarian stroma, accompanied by increased fibrosis. However, the Pearson analysis revealed that within the POI group, there was no significant correlation among fibrosis area, CD206^+^ M2-like MΦs density and the levels of key pro-inflammatory cytokines (IL-1β, IL-6 and TNF-α) in ovaries.

**Conclusions:**

Our descriptive study indicates that exacerbated inflammation and stromal fibrosis, as well as increased CD11c^+^ M1-like MΦs polarization, occur in mouse ovaries during chemotherapy. Since these are associative findings, future interventional studies including MΦs depletion are required to examine whether a causal relationship exists between CD11c^+^ M1-like MΦs and ovarian damage or between CD206^+^ M2-like MΦs and ovarian fibrosis in chemotherapy-induced POI. Moreover, additional functional research is necessary to determine whether modulating ovarian inflammation and M1/M2-like MΦs polarization would ameliorate chemotherapy-induced POI.

## Background

Recent years have seen an increasing trend of cancer occurring at a younger age in women (1, 2). Chemotherapy is commonly used for anti-cancer treatment (3, 4). Although this method has improved the patients’ survival rates, there is a growing concern about the chemotherapy-associated damage to ovarian function, particularly in patients of reproductive age (4, 5). This is because the ovaries are highly susceptible to the effects of chemotherapeutic drugs (4, 6). The chemotherapy can adversely affect the ovarian follicle reserve, ultimately leading to premature ovarian insufficiency (POI) and infertility (3, 5).

Notably, POI is a refractory disorder that impacts 1-5% of women of reproductive age (7). It is defined by the early onset of ovarian dysfunction in women under 40 years of age. POI often presents with menstrual disorders, including amenorrhea or oligomenorrhea, as well as hormonal abnormalities characterized by elevated follicle-stimulating hormone (FSH) and declined estradiol (E_2_) (8, 9). Additionally, given the positive association between anti-Müllerian hormone (AMH) and follicular reserve, recent studies have revealed that AMH also has the potential for POI diagnosis, with declined AMH levels in women with POI (10). Ultimately, POI can result in ovarian failure, female infertility, osteoporosis, cardiovascular disease, and even neurodegenerative disorders (7, 8). With the widespread use of chemotherapy in anti-cancer treatment, chemotherapy-induced POI is becoming a feared health issue that presents an increasing socioeconomic burden for worldwide communities (2, 9). However, until now, there is no available treatment to cure POI patients caused by chemotherapeutic drugs (5, 11). This highlights the urgent need to unravel the mechanisms underlying chemotherapy-induced POI development. It can, therefore, be beneficial in creating innovative strategies for this disease.

Although the pathogenesis of chemotherapy-induced POI remains unclear, previous research has reported the direct effects of chemotherapeutic drugs on ovarian follicle reserve, such as DNA damage, oxidative stress, follicle atresia and granulosa cells (GCs) apoptosis (2, 3, 5, 12). In addition, recent studies have revealed that chemotherapy also has the potential to disrupt homeostasis of the ovarian immune microenvironment by inducing inflammation and infiltration of immune cells within the ovary, ultimately leading to additional damage to ovarian function and oocyte development (13–15). Nevertheless, our understanding of the alterations in the ovarian immune microenvironment, including inflammatory status and immune cells, during chemotherapy is currently lacking.

It is known that tissue inflammation is primarily initiated by innate immune cells (16). Macrophages (MΦs) constitute a predominant population of innate immune cells in mammalian ovaries (17). They are characterized by high levels of heterogeneity and plasticity. In response to microenvironmental stimuli, they can polarize to an M1-like (classically activated) or M2-like (alternatively activated) phenotype (18–21). It has been revealed that MΦs play essential roles in inflammation, infection and injury, while also maintaining tissue homeostasis (17, 22). Generally, M1-like MΦs possess a pro-inflammatory phenotype and specialize in elimination of pathogens and injured cells (17, 23). Contrarily, M2-like MΦs possess an anti-inflammatory phenotype and primarily function in extracellular matrix remodeling and tissue repair (24, 25). Upon exposure to chemotherapeutic drugs, it is intriguing whether the M1/M2-like MΦs’ phenotype changes within ovarian tissues. However, our knowledge on these issues is still barren.

In this study, we aimed to evaluate the inflammatory status and investigate the alterations of the M1/M2-like MΦs’ phenotype within the mouse ovary during chemotherapy. To achieve this goal, we first established a POI mouse model with cyclophosphamide (CTX) and busulfan (BUS), followed by examining the effect of chemotherapeutic drugs on ovarian function and follicular reserve. Then, we assessed the systemic and local ovarian inflammatory status using the POI model. Finally, we examined the alterations of ovarian MΦs populations during chemotherapy-induced POI.

## Materials and methods

### Establishment of POI mouse models and ethical approval

Eight-week-old female C57BL/6 mice were purchased from and housed in a temperature (22 ± 2 °C)- and humidity (60%-65%)-controlled barrier facility at the Daoke Medical & Pharmaceutical Company (Guangzhou, China) under a 12-hour light/dark cycle, with free access to mouse chow and water. All mice were acclimatized for one week. All animal experiments were approved by the Animal Ethics Committee of the Guangdong Provincial People’s Hospital (ECD No. KY-Z-2022-379-01), Southern Medical University, Guangzhou, China. Humane endpoints were clearly defined and strictly followed throughout the experiments.

To establish a mouse POI model, we referred to the reported drug doses in previous studies (26–29). To promptly select a relatively suitable dose while reducing the number of mice used, we set up and tested a narrow range of different combined doses of CTX (Sigma-Aldrich) and BUS (Sigma-Aldrich), as shown below. The sample size was determined based on power analysis and referred to prior literature (27, 30). Briefly, the primary metric utilized in our calculation was the anticipated difference in the number of primordial follicles between the treatment and control groups, with the significance level (α) set at 0.05 and the statistical power set at 0.8. The results of the calculation indicated that a minimum of 7 mice per group was required. Furthermore, we incorporated a 10% potential redundancy margin for each group, and the final sample size was adjusted to 8 mice per group. Accordingly, forty C57BL/6 female mice were randomly allocated into five groups (n=8 mice per group), each receiving a single intraperitoneal (IP) injection of varying doses of CTX and BUS, except the control group. Group I received 90 mg/kg of CTX and 15 mg/kg of BUS. Group II received 120 mg/kg of CTX and 15 mg/kg of BUS. Group III received 150 mg/kg of CTX and 15 mg/kg of BUS. Group IV was administered 150 mg/kg of CTX and 30 mg/kg of BUS. The controls received an IP injection of an equal volume of PBS. After two weeks, the serum and ovaries were obtained to further assess the feasibility of the POI mouse model. The comprehensive procedure is shown in the schematic design of the animal experiment (**Figure 1A**). The sacrifice method used in mice was cervical dislocation by skilled investigators, a method employed for the swift euthanasia of the animals. To avoid the bias, the above dose-ranging study was first performed as a separate cohort to identify a relatively suitable CTX/BUS dose (see the first part of the results: 150 mg/kg CTX and 15 mg/kg BUS, Group III). Then, we initiated a new independent cohort that received the selected Group III dose or PBS control and was used for the subsequent main experiments, including inflammation, flow cytometry, immunofluorescence, immunohistochemistry and fibrosis. No samples from the initial dose-ranging study mice were carried over into the subsequent main experiments.

**Figure 1:**
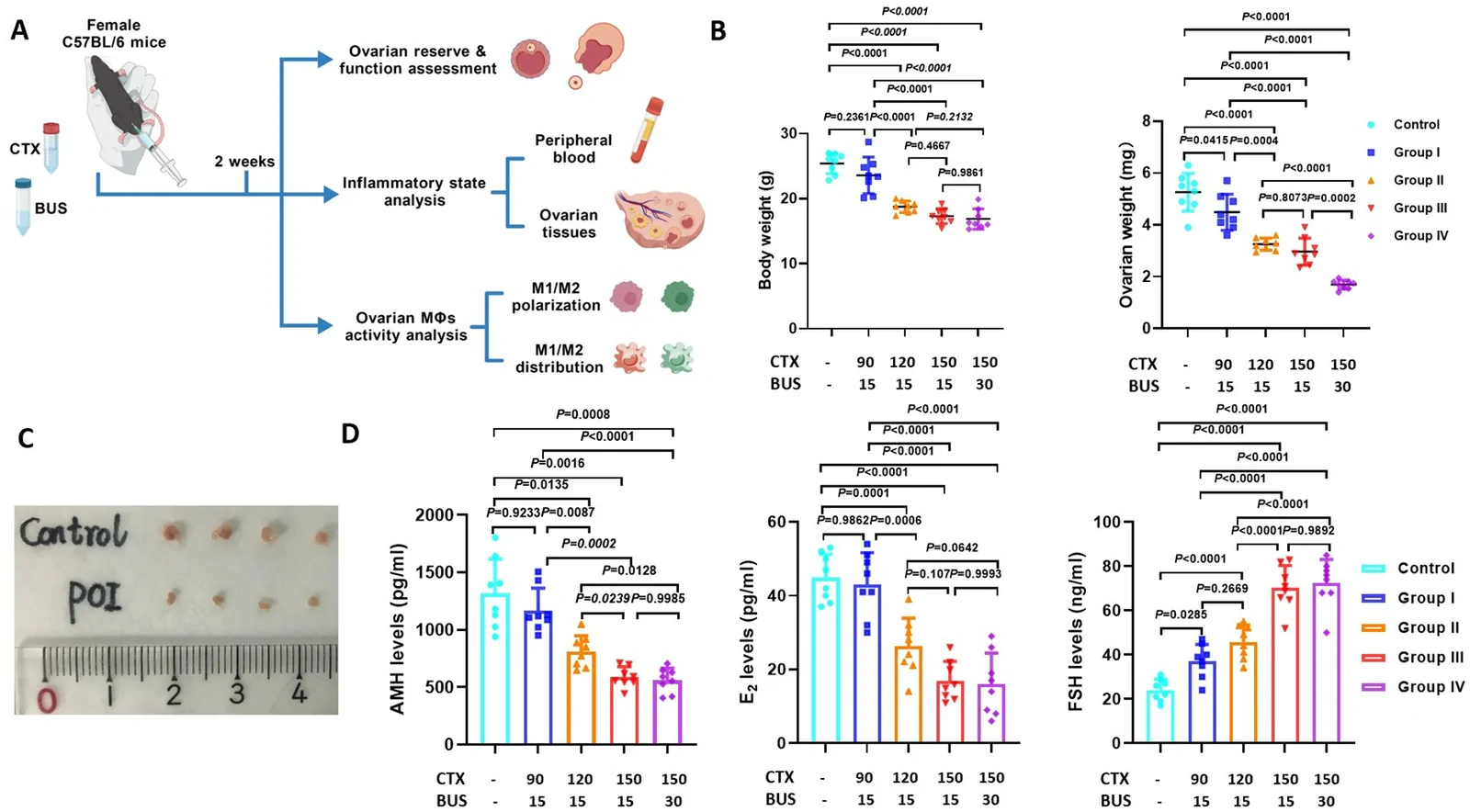
Effects of CTX and BUS on general conditions and sex hormone levels in mice. **(A)** Schematic design of the animal experiments. **(B)** The body weights and ovarian weights of mice were examined two weeks after exposure to CTX/BUS. n=8 mice per group, and 3 independent experiments were performed. **(C)** Representative images of mouse ovaries from the control and Groups III. **(D)** The levels of AMH, E_2_ and FSH in the serum from each group were examined by ELISA at two weeks after exposure to CTX/BUS. n=8 mice per group, and 3 independent experiments were performed. Mice in Group I, II, III and IV received CTX/BUS at 90/15 mg/kg, 120/15 mg/kg, 150/15 mg/kg and 150/30 mg/kg, respectively. The controls received an equal volume of PBS. Data are represented as the mean ± SD. Among the groups, an ANOVA with Tukey’s Multiple Comparison Test was performed except for AMH levels (Welch’s ANOVA with Dunnett’s T3 multiple comparisons test).

### Assessment of ovarian reserve

The mouse ovaries were fixed with 4% paraformaldehyde overnight and thereafter embedded in paraffin. The whole ovary was serially sectioned at a thickness of 5 μm and stained with hematoxylin and eosin (H&E). Ovarian follicle quantification was performed on these H&E-stained sections using a light microscope at ×40, ×100 and ×400 final magnifications (OLYMPUS, CX31), as previously described with minor modifications (31–33). To avoid counting a follicle twice, the raw number of follicles at various stages was tallied in every fifth serial section throughout the entire ovary from a random start. All selected sections were evaluated per ovary. To maintain reproducibility and accuracy, only the follicles with a clear oocyte nucleus were counted. The enumeration covered the entire ovarian area of each section, commencing from a standardized 12 o’clock position and progressing clockwise. Finally, the total follicle count per ovary was estimated by multiplying the raw counts by a correction factor of five to account for the uncounted sections (33, 34). This correction factor accounts for the sampling interval (every fifth section) and corrects for counting only follicles with a visible nucleus. As a follicle can be present in more than one 5 μm sections, this factor translates the raw count into an estimate of the actual number of follicles in the entire ovary based on the established principles of stereology (33, 34).

The criteria used for classifying ovarian follicles were based on their morphological characteristics (31, 32). Primordial follicles were characterized as having a partial or complete layer of flattened GCs surrounding a compact oocyte. Primary follicles were identified by a single layer of cuboidal GCs surrounding an enlarged oocyte. Secondary follicles were defined as having more than one layer of cuboidal GCs surrounding an enlarged oocyte, without a visible antrum. Antral follicles showed the areas of follicular fluid (antrum) or a single large antral space. Atretic follicles were identified by a degenerating process (convoluted and condensed or fragmented) without ovulation. To ensure objectivity, all follicle counting was carried out by two independent investigators who were blinded to the origin of the sections.

### Mouse oocyte recovery

Oocytes were retrieved and cultured as previously described (35). Briefly, C57BL/6 female mice were hyperstimulated with pregnant mare’s serum gonadotrophin (PMSG, 7.5 IU) followed by human chorionic gonadotropin (hCG, 7.5 IU) 46–48 h later. Subsequently, Metaphase II (MII) oocytes were collected from females 12–14 h after hCG injection. The collection procedures were conducted in HEPES-buffered potassium simplex optimized medium (KSOM-HEPES) with the addition of 4 mg/ml bovine serum albumin (BSA, Sigma-Aldrich). Cumulus cells around the oocytes were removed by a brief exposure to 200 IU/ml hyaluronidase in KSOM-HEPES. Given that the morphology of oocytes is reportedly associated with oocyte quality (36, 37), all oocytes were then kept in KSOM medium under mineral oil at 37 °C in 6% CO_2_ and 5% O_2_ conditions (within 1 h) until morphological evaluation.

### Quantitative real-time polymerase chain reaction

Total RNA was extracted from mouse ovaries using TRIzol reagent (Servicebio). All RNA samples were examined by pre-defined sample quality control (QC) criteria based on RNA purity (A260/A280: 1.8-2.0), RNA concentration (≥ 200 ng/μL) and integrity (RNA Integrity Number ≥ 7.0). Only samples meeting the QC criteria were included; otherwise, they were excluded before downstream analysis and were not replaced in order to preserve the independence of biological replicates. Then, cDNA was synthesized using a SweScript All-in-One RT SuperMix for qPCR kit (Servicebio, Cat# G3337) as per the manufacturer’s instructions. The reverse transcription was performed using the thermal conditions (25 °C for 5 min; 42 °C for 30 min; 85 °C for 5 s). Next, RT-qPCR was performed using the Universal Blue SYBR Green qPCR Master Mix kit (Servicebio, Cat# G3326) on a Real-Time PCR system (CFX Connect, Bio-Rad). The 15 μl qPCR reaction mixture included 2×Universal Blue SYBR Green qPCR Master Mix (7.5 μl), forward and reverse primers (1.5 μl), cDNA (2.0 μl) and nuclease-free water (4.0 μl). Finally, the PCR reaction program consisted of pre-denaturation at 95 °C for 30 s, followed by 40 cycles of denaturation at 95 °C for 15 s and annealing-extension at 60 °C for 30 s, concluded by a melting curve analysis (65 °C to 95 °C). Each sample was analyzed in triplicate (three technical replicates). The relative expression levels of target genes were calculated using the 2^-ΔΔ^*^C^_T_* method normalized to glyceraldehyde 3-phosphate dehydrogenase (*Gapdh*), where ΔΔ*C_T_* = (*C_T, target_*_−_ *C_T, Gapdh_*) _treated sample −_ (*C_T, target_*_−_*C_T, Gapdh_*) _control sample._ All RT-qPCR primer pairs are listed in **Table 1**.

**Table 1:** Real-Time PCR primers.

Gene	Forward primer 5’-3’	Reverse primer 5’-3’
*Il1a*	GGACAAACACTATCTCAGCACCAC	TCTGGCAACTCCTTCAGCAAC
*Il1b*	GCATCCAGCTTCAAATCTCGC	TGTTCATCTCGGAGCCTGTAGTG
*Il6*	CCCCAATTTCCAATGCTCTCC	CGCACTAGGTTTGCCGAGTA
*Tnfa*	CCCTCACACTCACAAACCACC	CTTTGAGATCCATGCCGTTG
*Il4*	CAGCTAGTTGTCATCCTGCTCTTC	TCCCTTCTCCTGTGACCTCGTT
*Il10*	TTTAAGGGTTACTTGGGTTGCC	AATGCTCCTTGATTTCTGGGC
*Il13*	AGGACCCAGAGGATATTGCATG	CCGTGGCGAAACAGTTGCT
*Gapdh*	CCTCGTCCCGTAGACAAAATG	TGAGGTCAATGAAGGGGTCGT

### Enzyme-linked immunosorbent assay

The blood samples were collected from the female mice by retro-orbital puncture under anesthesia and were then preserved for 2 h at room temperature (RT). Subsequently, the samples were centrifuged at 3000 rpm for 15 min to extract the serum. Only serum samples meeting the pre-defined sample quality and the sample volume requirements as per the ELISA kits’ instructions were included; otherwise, they were excluded before downstream analysis and were not replaced to preserve the independence of biological replicates. Next, the concentrations of anti-Müllerian hormone (AMH), E_2_, and FSH in serum were analyzed using specific murine ELISA kits (Cloud-Clone Corp, CEA228Mu (AMH), CEA461Ge (E_2_) and SEA830Mu (FSH)) according to the manufacturer’s instructions. In addition, the levels of interleukin-6 (IL-6), interleukin-18 (IL-18) and tumor necrosis factor-α (TNF-α) in serum were measured by mouse ELISA kits (R&D Systems, M6000B (IL-6), DY7625 (IL-18) and DY410 (TNF-α)) based on the manufacturer’s instructions. Both standards and samples were assayed in duplicates. Finally, the absorbance was determined at 450 nm using the microplate reader (BioTeK, Epoch).

### Ovarian cell isolation

The fresh ovaries were placed in ice-cold RPMI 1640 (Sigma-Aldrich, R8758) immediately after the sacrifice. Then, ovarian tissues were dissected and minced carefully using microsurgical scissors. The fragmented tissues were digested in RPMI-1640 supplemented with 5% FBS, containing 20μg/ml deoxyribonuclease I (Sigma-Aldrich, DN25), 1.5mg/ml collagenase IV (Sigma-Aldrich, C5138), 6mg/ml dispase II (Sigma-Aldrich, D4693) and 1.5mg/ml hyaluronidase (Sigma-Aldrich, H3506) for 30 min at 37 °C. Next, digestion was halted with the addition of 3ml HBSS containing 5% FBS and 5mM EDTA. The suspension was filtered through a 40 μm nylon mesh filter. Finally, the cell pellet was obtained following centrifugation (500×g for 5 min at 4 °C) and was washed with HBSS.

### Flow cytometric analysis

Ovarian cell suspensions were initially counted and stained with LIVE/DEAD Fixable Dead Cell Stains (Fixable Viability Stain 780, BD, 565388) for 30 min at 4 °C. After washes with FACS buffer (HBSS containing 3% FBS), the cells were blocked with Rat Anti-Mouse CD16/CD32 (Mouse BD Fc Block™) for 5 min at 4 °C. Then, for surface staining, cells were subsequently incubated with PerCP-Cy5.5 Rat Anti-Mouse CD45 (BD, 561869), PE Rat Anti-Mouse F4/80 (BD, 565410), FITC anti-mouse CD11c (Biolegend, 117305) and APC anti-mouse CD206 (Biolegend, No. 141707) for 20 min at 4 °C in the dark. Next, after several washes with FACS buffer and centrifugation (500×g for 5 min at 4 °C), cells were resuspended and fixed with 1% paraformaldehyde (PFA). Lastly, samples were examined on an LSRFortessa flow cytometer (BD Biosciences) with application settings. Data was analyzed using FlowJo software (Version 9.9.4, Treestar, Ashland, OR, USA). The gating strategy for the characterization of ovarian MΦs phenotypes is described in **Figure 2A**. To avoid the bias, the investigators were blinded to the origin of the samples.

**Figure 2:**
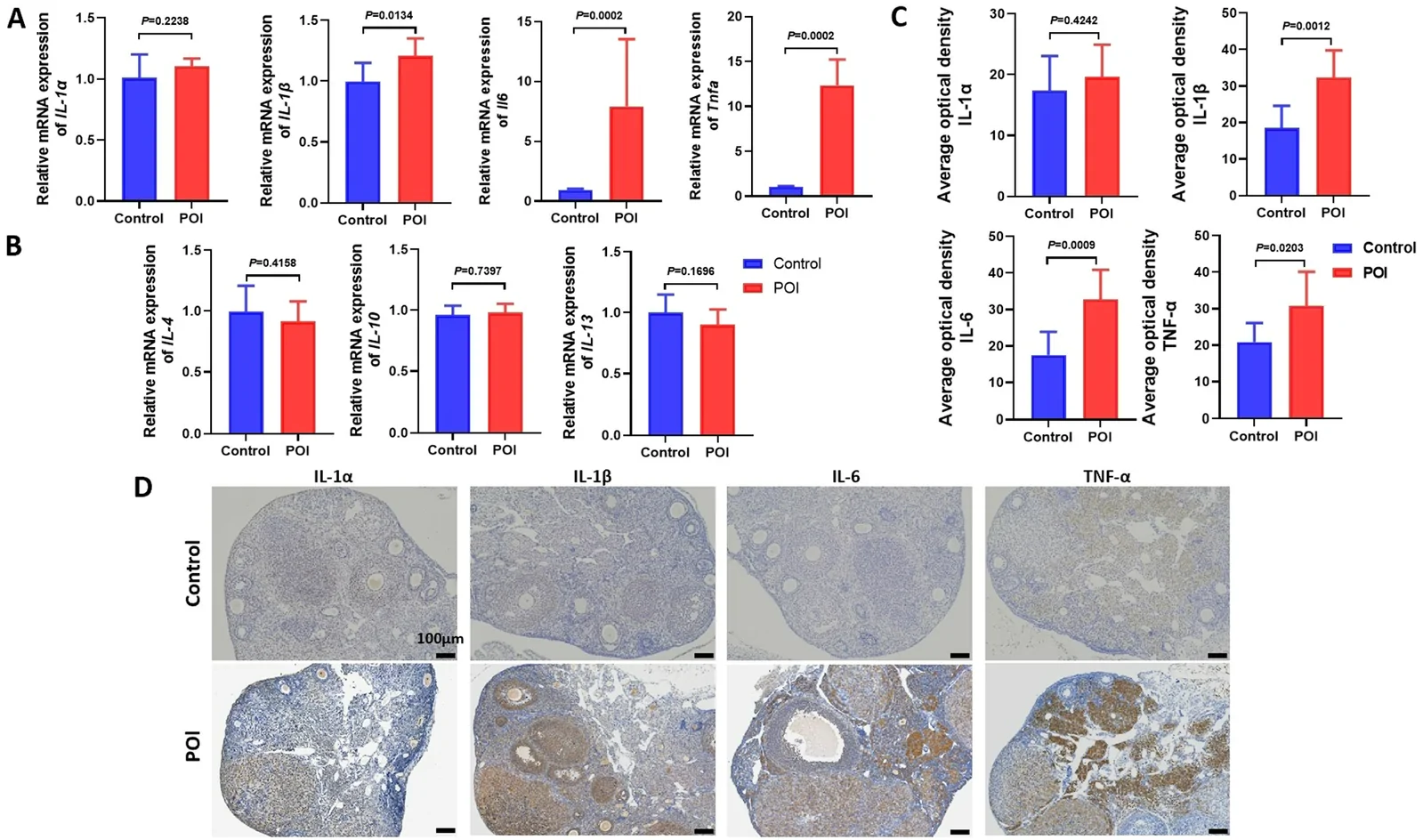
Analysis of M1/M2 macrophages in mouse ovaries along with CTX/BUS treatment using flow cytometry. **(A)** Gating strategy for the determination of M1-like and M2-like MΦs within ovaries. Briefly, ovarian cells were first gated on a forward (FSC-A) and side light scatter (SSC-A) plot. Then, taking only live cells, M1-like MΦs were gated as CD45^+^ F4/80^+^ CD11c^+^ CD206^-^ cells, whereas ovarian M2-like MΦs gated as CD45^+^ F4/80^+^ CD11c^-^ CD206^+^ cells. **(B)** The proportions of M1-like and M2-like MΦs in ovaries obtained from control and CTX/BUS-treated mice. Each symbol represents one mouse. n=8 mice per group, and 3 independent experiments were performed. Data are represented as the mean ± SD. To avoid the bias, the investigators were blinded to the group assignments of the samples. Between the groups, an unpaired *t*-test with Welch’s correction was performed on the proportions of the M1-like MΦs comparison, and an unpaired Student’s *t*-test was performed on the proportions of the M2-like MΦs comparison.

### Immunofluorescence staining

The mouse ovary was serially sectioned at a thickness of 5 μm. The sections were deparaffinized and rehydrated, and antigen retrieval was performed at high temperature (95 °C) in PBS (pH 7.4) for three times. Then, endogenous peroxidase activity of ovarian sections was blocked in 3% hydrogen peroxide for 25 min at RT. After blocking with BSA, the sections were stained with primary antibodies overnight at 4 °C. Next, following washes, the sections were incubated with secondary antibodies for 50 min at RT. Nuclear DNA was then stained with DAPI (Servicebio, China) for 10 min at RT. All antibodies were purchased from Servicebio Technology Co. (China) and listed with dilutions in **Supplementary Table 2**. To ensure the specificity of IF staining, spleen sections from female C57BL/6 mice served as a positive control for F4/80, CD11c and CD206, while uterine sections served as a positive control for α-SMA. For negative controls, the ovarian sections were subjected to: 1) isotype controls, where primary antibodies were replaced with isotype-matched control IgG at equivalent concentrations; and 2) omission controls, where primary antibodies were replaced with antibody dilution buffer (38). Subsequently, both control groups were incubated with the corresponding secondary antibodies under the same experimental conditions. Finally, the sections were mounted, and images were obtained using a fluorescence microscopy (NIKON ECLIPSE C1).

Both ovaries were obtained from each mouse. For each ovary, to avoid double-counting the same macrophage, two non-consecutive representative sections with an interval of 40 μm from the largest cross-section through the ovarian center were selected for analysis (20). To avoid regional bias, six non-overlapping fields were selected across each section at a high-power field (HPF, ×400 magnification) using a systematic uniform random sampling method (39). Specifically, the region of interest (ROI) outlining the entire ovary section was first defined. Then, imaging areas were selected by moving the microscope stage in a systematic grid pattern across the ROI, capturing the first available field of view within each grid square. The mean fluorescence intensity per HPF was examined using Fiji ImageJ2 software. The density of CD206^+^ M2-like MΦs was assessed by Fiji ImageJ2 software and expressed as the average number of positive cells per HPF within the ovarian stroma. To avoid the bias, the investigators were blinded to the group assignments of the samples.

### Immunohistochemistry analysis

The IHC analysis was used to examine the protein expression levels of IL-1α, IL-1β, IL-6 and TNF-α in the mouse ovaries. Firstly, the ovary was serially sliced into 5 μm sections. Dewaxed and rehydrated sections were successively subjected to antigen retrieval, endogenous peroxidase activity blocking and serum sealing. Next, the sections were incubated with primary and secondary antibodies presented with dilutions in **Supplementary Table 3**, followed by staining with 3,3′-diaminobenzidine (DAB) (Servicebio, Cat# G1212) and hematoxylin (Servicebio, Cat# G1004). In parallel, negative controls were obtained by replacing the primary antibody with isotype-matched control IgG or antibody dilution buffer, referring to the aforementioned IF staining (38). Due to low expressions of IL-1α, IL-1β, IL-6 and TNF-α in normal mouse tissues, we included lipopolysaccharide (LPS)-induced C57BL/6 mouse spleen or liver (sampled 8 h post-stimulation) gifted by Daoke Medical & Pharmaceutical Company as positive controls (40, 41). After washes, the sections were sealed and imaged using a microscope (NIKON E100) installed with an LG-S80 digital slide scanner (Servicebio).

Both ovaries were harvested from each mouse. For each ovary, two consecutive representative sections from the largest cross-section through the ovarian center were chosen for analysis. Rather than manually selecting individual fields, entire ovary sections were digitally scanned​ using a high-resolution slide scanner (LG-S80). For each section, the entire ovarian cross-sectional imaging area​ was outlined, and Fiji ImageJ2 software was used to assess the average optical density. To avoid the bias, the investigators were blinded to the group assignments of the samples.

### Masson staining

The ovarian fibrosis was quantified by Masson-stain using a Masson dye solution set (Servicebio, China) according to the manufacturer’s instructions. Briefly, ovarian sections were deparaffinized with Xylene I/II, 100% ethanol I/II and 75% ethanol. After a wash with tap water, the slices were soaked in Masson A overnight and rinsed with tap water. Then, the slices were stained with Masson solution (Masson B and C mix) for 1 min followed by the differentiation with 1% hydrochloric acid alcohol. Following a rinse in tap water, the slices were soaked in Masson D for 6 min, Masson E for 1 min, and Masson F for 2–30 s. Subsequently, the slices were washed with 1% glacial acetic acid and then dehydrated using two cups of anhydrous ethanol. Next, the slices were transferred to 100% ethanol for 5 min and xylene for 5 min. Finally, the slices were sealed with neutral gum. Regions of fibrosis, denoted in blue, were quantified using Fiji ImageJ2 software. To avoid the bias, the investigators were blinded to the group assignments of the samples.

### Statistical analysis

All statistical analyses were conducted using GraphPad Prism software (v9.00, GraphPad, San Diego, CA, USA) and SPSS (version 23.0; SPSS Inc.). The Shapiro-Wilk normality test was used to evaluate the normality of data distribution. Then, homogeneity of variance was assessed using a Bartlett’s test or Brown-Forsythe test. If data were normally distributed and group variances were equal, an ANOVA with Tukey’s multiple comparison test (for multiple groups) or Student’s *t*-test (for two groups) was used. When data were normally distributed but group variances were unequal, Welch’s ANOVA with Dunnett’s T3 multiple comparisons test (for multiple groups) or Welch’s *t*-test (for two groups) was conducted. When data were not normally distributed, for two-group comparisons, a Mann-Whitney *U*-test (for independent samples) was performed. The continuous variables were shown as mean and standard deviation (mean ± SD) or median with interquartile range (IQR) as appropriate. The correlation between fibrosis area, CD206^+^ M2-like MΦs density and the levels of key pro-inflammatory cytokines was evaluated by Pearson analysis. Additionally, variations in sample sizes across assays were attributable to independent experimental replication (ELISA) or pre-defined sample QC criteria (qPCR), not *post-hoc* exclusions. *P*<0.05 was considered statistically significant.

## Results

### Ovarian function and reserve decline in chemotherapy-induced POI mice

To characterize the progression of ovarian dysfunction during chemotherapy-induced POI, the female mice were given a single IP injection of different doses of CTX and BUS, followed by the analysis of various markers (**Figure 1A**). Firstly, we assessed the general condition of control and mouse models. The mortality, adverse effects and body weights were assessed bi-daily, whereas the ovarian weights were evaluated two weeks after chemotherapy. The initial body weights of all the mice are presented in **Supplementary Table 1**. There were no statistically significant differences in initial body weights among the five groups (**Supplementary Table 1**). No mortality was found in all treated mice. Among all groups, only in Group IV, five out of eight mice showed signs of adverse effects, including reduced mobility, lethargy and alopecia, but did not reach the humane endpoint criteria and all survived to the scheduled endpoint. Data from these symptomatic mice were included in the final analysis to avoid bias. These findings indicate the safety and tolerability of the different dosing regimens used in Group I, II and III. Moreover, in comparison to the controls, the body and ovarian weights of mice in Groups II, III and IV exhibited a considerable reduction (**Figures 1B, C**). Secondly, the sex hormone analysis revealed that compared with the controls, the levels of AMH in Groups II, III and IV were decreased (*P=*0.0135, *P* = 0.0016 and *P* = 0.0008, respectively; **Figure 1D**); the E_2_ levels in Groups II, III and IV were dramatically reduced (*P=*0.0001, *P*<0.0001 and *P*<0.0001, respectively), whereas FSH exhibited a marked rise in Groups I, II, III and IV (*P=*0.0285, *P*<0.0001, *P*<0.0001 and *P*<0.0001, respectively; **Figure 1D**). Ultimately, we further adopted the number of developing follicles, including primordial, primary, secondary and antral follicles, as a parameter to assess the ovarian reserve. Histological analysis of ovaries revealed a progressive decline in the number of growing follicles in Groups I, II, III and IV as compared to the controls (**Figures 3A, B**; **Supplementary Figure 1**). In contrast, the number of atretic follicles in the ovaries exhibits an opposing alteration (**Figure 3B**). Furthermore, following superovulation, oocyte yield reduced considerably in the four treatment groups compared to the controls, particularly in Groups III and IV (all *P*<0.0001; **Figure 3C**). Nevertheless, the morphology of MII oocytes was comparable among these groups (**Figure 3D**). The findings indicate that ovarian damage progressively intensified with escalating doses of CTX and BUS in mice, characterized by a gradual decrease in both ovarian reserve and the number of ovulated oocytes. However, when the doses were increased to the highest levels in Group IV, no significant difference was seen between Group III and Group IV regarding follicle counts and hormone levels (**Figure 1D**; **Figures 3B, C**). To reduce the doses used in mice, we chose Group III (150 mg/kg CTX and 15 mg/kg BUS) to create the POI model.

**Figure 3:**
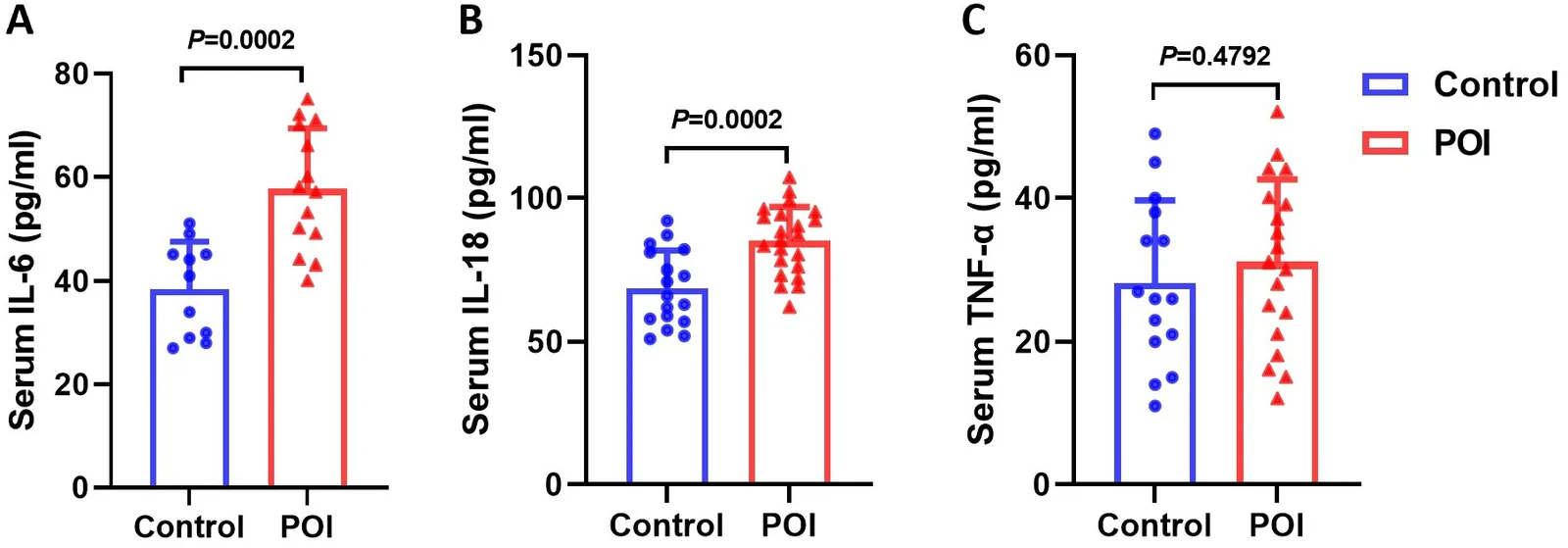
Effects of CTX and BUS on the ovarian reserve in mice. **(A)** Representative H&E staining images of histological ovarian sections in the controls and Group III, showing primordial (o), primary (*), secondary (★), antral (arrowhead) and atretic follicles (arrow). The left panels (×100 magnification) and right panels (×400 magnification) represent the magnified image of black frames. Scale bar: 100μm (left) and 50μm (right). **(B)** The quantification of different stages of ovarian follicles per ovary in the controls and CTX/BUS-treated groups. n=8 ovaries from 8 mice per group, and 3 independent experiments were performed. The number of sections used for counting follicles per ovary for each group was as follows: 57 (control), 52 (Group I), 40 (Group II), 38 (Group III), 31 (Group IV). **(C)** The quantification of ovulated oocytes collected after hormonal stimulation of the controls and CTX/BUS-treated mice. n=8 mice per group, and 3 independent experiments were performed. **(D)** Representative morphological images (×200 magnification) of ovulated oocytes from the controls and Group III. Scale bar: 50μm. Data are represented as the mean ± SD. To avoid the bias, the investigators were blinded to the group assignments of the samples. Among the groups, a Welch’s ANOVA with Dunnett’s T3 multiple comparisons test was performed on the data from **(B)** (primordial follicles and primary follicles), and an ANOVA with Tukey’s Multiple Comparison Test was performed on the rest of the data from **(B, C)**.

### The mouse model receiving chemotherapy exhibits an inflammatory microenvironment in the ovary

It is indicated that chemotherapy-induced POI might be associated with a gradual shift towards a pro-inflammatory cytokine profile. To explore the correlation between ovarian injury and intensified pro-inflammatory state, we analyzed the mRNA expression levels of key pro-inflammatory genes including interleukin-1α (*Il1a*), interleukin-1β (*Il1b*)*, Il6* and *Tnfa* in the ovaries of chemotherapy-induced POI mice by qPCR (**Figure 4A**). The results revealed a discernible pattern of increased expression levels for *Il1b, Il6* and *Tnfa* during POI progression. They showed markedly elevated levels in the ovaries of chemotherapy-induced POI mice in comparison to those of controls (*P=*0.0134, *P* = 0.0022 and *P* = 0.0022, respectively) (**Figure 4A**). Nevertheless, there was no significant difference in *Il1a* levels between POI and control groups (*P* = 0.2238; **Figure 4A**). Consistently, IHC results showed that except for IL-1α, the levels of IL-1β, IL-6 and TNF-α were significantly higher in the POI group than those of the controls (*P* = 0.4242, *P* = 0.0012, *P* = 0.0009 and *P* = 0.0203, respectively; **Figures 4C, D**). In parallel, we also examined the anti-inflammatory cytokines, including interleukin-4 (*Il4*), interleukin-10 (*Il10*) and interleukin-13 (*Il13*) in the ovaries of chemotherapy-induced POI mice using qPCR. We observed no significant difference in the levels of these cytokines between POI and control groups (*P* = 0.4158, *P* = 0.7397 and *P* = 0.1696, respectively; **Figure 4B**). Overall, these findings suggest an inflammatory microenvironment in the ovaries of chemotherapy-induced POI mice, coinciding with ovarian functional decay and follicle pool decline.

**Figure 4:**
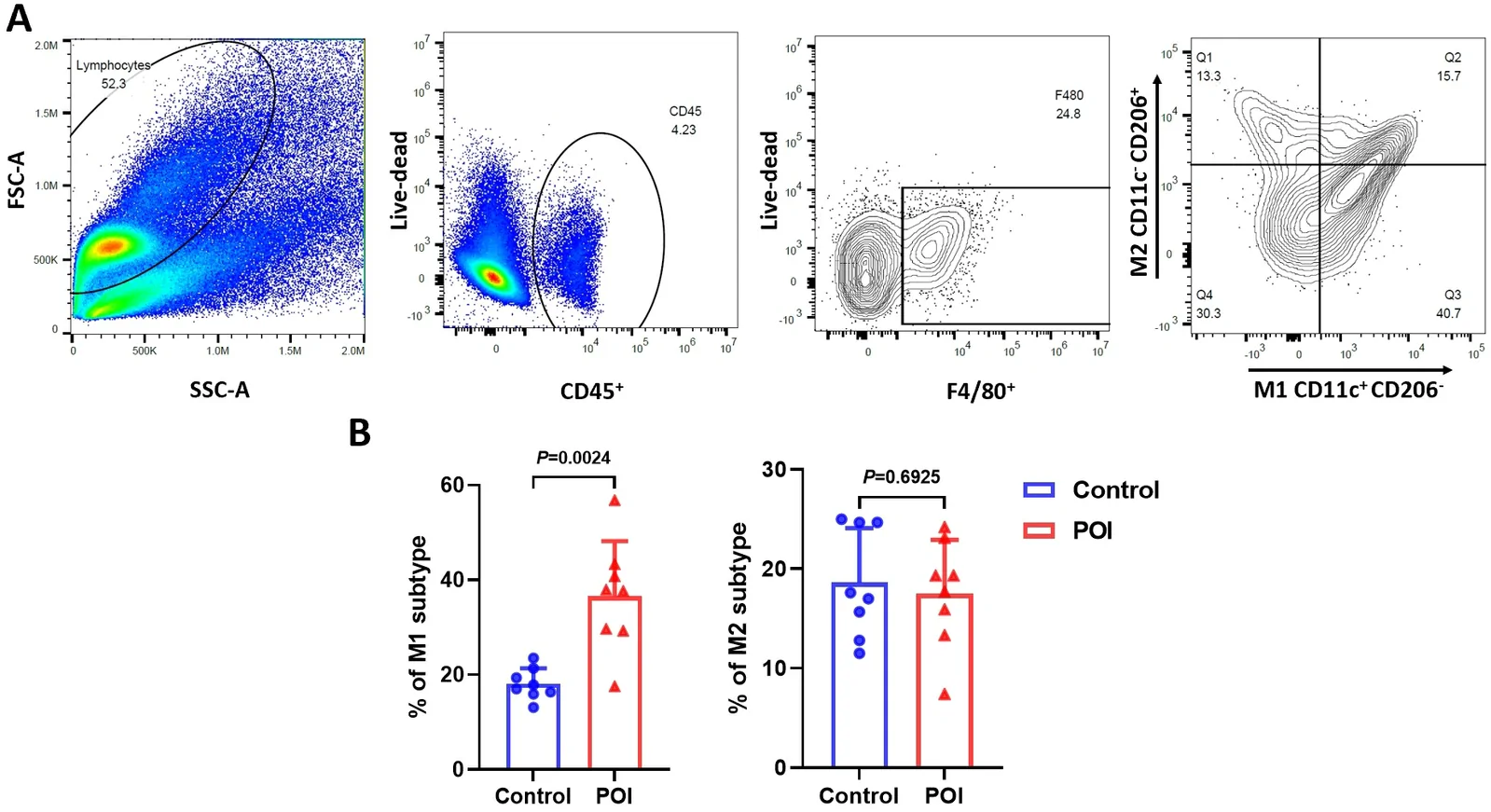
Chemotherapy-induced POI is associated with a local inflammatory microenvironment in mouse ovaries. **(A)** Relative mRNA expression levels of *Il1a*, *Il1b*, *Il6* and *Tnfa* in ovaries obtained from the controls and CTX/BUS-treated mice. For *Il1a* and *Il1b*, n=8 mice per group, and 3 independent experiments were performed. For *Il6* and *Tnfa*, n=6 mice per group, and 3 independent experiments were performed. **(B)** Relative mRNA expression levels of *Il4*, *Il10* and *Il13* in ovaries obtained from control and CTX/BUS treated mice. n=8 mice per group, and 3 independent experiments were performed. For **(A, B)**, n represents biologically independent animals whose RNA quality met pre-defined sample QC criteria. Regarding the unequal sample sizes in **(A)**, for each of *Il6* and *Tnfa*, some extracted RNA samples from both control (n=2) and POI (n=2) groups failing to meet pre-defined QC were excluded before downstream analysis. **(C)** IHC analysis of *Il1α*, *Il1β*, *Il6* and *Tnfα* expressions in each group. n=8 mice per group, and 3 independent experiments were performed. **(D)** Representative IHC images (×100 magnification) of IL-1α, IL-1β, IL-6 and TNF-α expressions in each group. Scale bar: 100μm. Data are represented as the mean ± SD, except for **(A, B)** (*Il1a*, *Il6*, *Tnfa* and *Il10*) presenting with the median with IQR. For data from **(A, B)**, between the groups, an unpaired Student’s *t*-test was performed, except for *Il1a*, *Il6*, *Tnfa* and *Il10* (Mann-Whitney *U*-test; *U* = 20, *U* = 0, *U* = 0 and *U* = 28.5, respectively). For data from **(C)**, an unpaired Student’s *t*-test was performed. To avoid the bias, the investigators were blinded to the group assignments of the samples.

### Chemotherapy-induced POI is associated with elevated serum levels of the pro-inflammatory cytokines IL-6 and IL-18 in mice

In addition to the ovarian local environment, we further evaluated the systemic inflammatory condition during chemotherapy-induced POI in mouse models. The concentrations of pro-inflammatory cytokines involving IL-6, IL-18, and TNF-α were quantified in the serum obtained from chemotherapy-induced POI mice and controls using ELISA (**Figure 5**). Our findings indicated that the serum levels of both IL-6 and IL-18 were considerably elevated in chemotherapy-induced POI mice compared to the controls (both *P* = 0.0002; **Figures 5A, B**). Nevertheless, the serum TNF-α did not show significant alterations during POI progression (*P* = 0.4792; **Figure 5C**). Together, our results indicate heightened systemic inflammation, as reflected by the increased levels of IL-6 and IL-18 in peripheral circulation along with the development of POI induced by chemotherapeutic agents.

**Figure 5:**
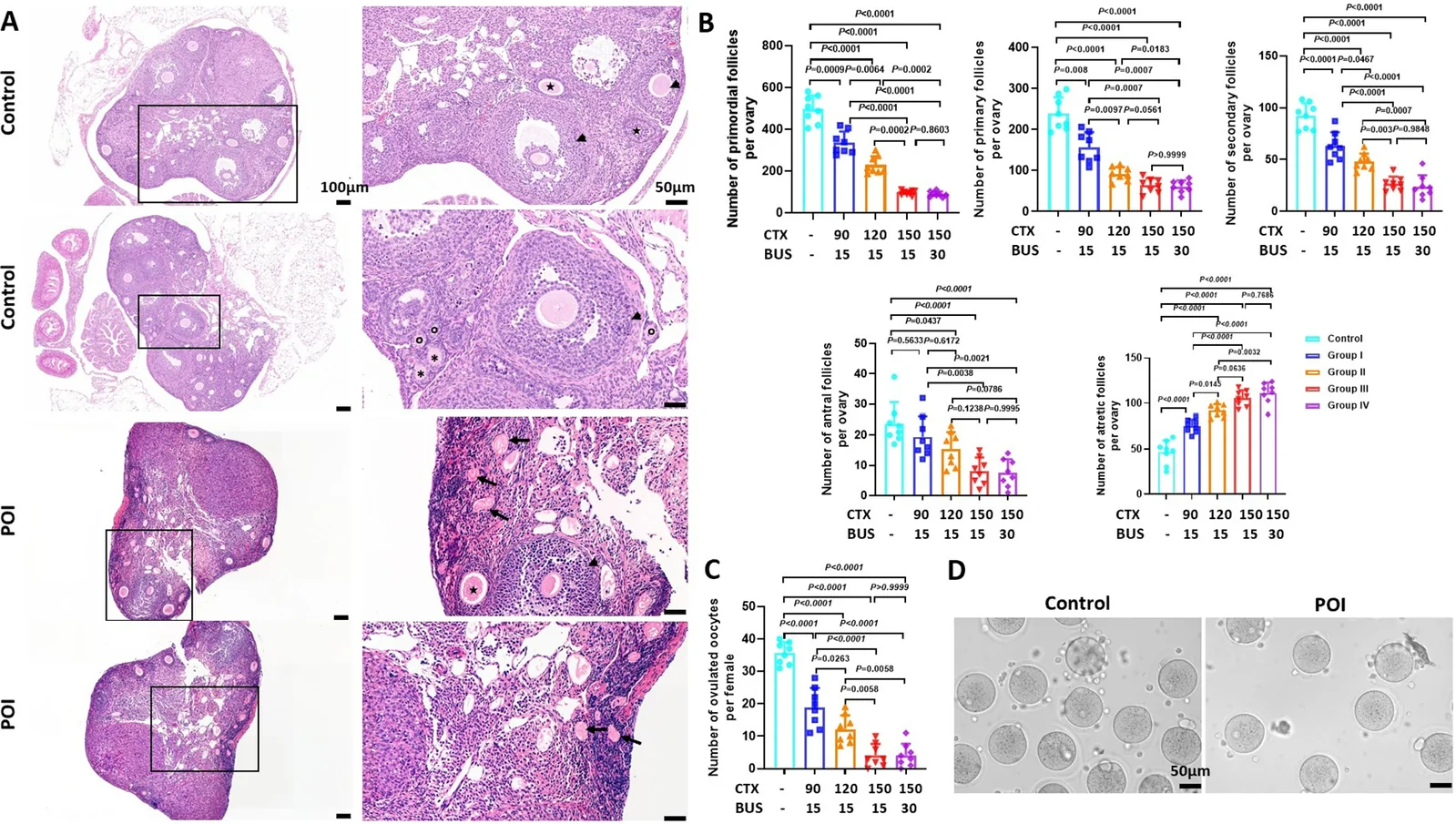
Chemotherapy-induced POI is associated with elevated serum levels of the pro-inflammatory cytokines in mice. **(A–C)** Serum concentrations of IL-6, IL-18, and TNF-α in control and CTX/BUS-treated mice. Each symbol represents one mouse. n=11–22 mice per group, and 3 independent experiments were performed; n represents biologically independent animals whose serum samples met the pre-defined ELISA sample quality requirements; the varying sample sizes reflect the real cumulative number of biologically independent animals across these three independent experimental cohorts. Data are represented as the mean ± SD. Between the groups, an unpaired Student’s *t*-test was performed. No data were excluded because of statistical outliers or technical failure.

### Dynamic alterations in polarization of M1/M2-like macrophages during chemotherapy-induced POI in mice

It is known that macrophages (MΦs) are the most abundant leucocytes within mammalian ovaries, and they play a critical role in folliculogenesis, ovulation and luteinization. Different MΦs subtypes perform diverse functions during inflammation. Considering that chemotherapy-induced POI is associated with the exaggerated systemic inflammation and inflammatory microenvironment within ovaries, we examined whether there are changes of the M1/M2-like MΦs’ phenotype in the ovary during POI progression using flow cytometry. The gating strategy was illustrated in **Figure 2A**. Briefly, ovarian M1-like MΦs were defined as CD45^+^ F4/80^+^ CD11c^+^ CD206^-^ cells, whereas ovarian M2-like MΦs were defined as CD45^+^ F4/80^+^ CD11c^-^ CD206^+^ cells (**Figure 2A**). The results showed that the proportion of CD11c^+^ M1-like MΦs was significantly increased in chemotherapy-induced POI mice compared to those of the controls (*P* = 0.0024; **Figure 2B**). By contrast, there was no significant difference in the proportion of CD206^+^ M2-like MΦs between the POI group and the controls (*P* = 0.6925; **Figure 2B**). Hence, these findings suggest an increased polarization of CD11c^+^ M1-like MΦs in mouse ovaries during chemotherapy-induced POI.

### Differential distribution of two polarized M1/M2-like macrophages within mouse ovaries after chemotherapy

Next, we further assessed the distribution of MΦs subtypes in ovaries of CTX/BUS-treated mouse models using immunofluorescence. MΦs were labelled with F4/80, while M1-like and M2-like MΦs were labelled with CD11c and CD206, respectively. The immunostaining results revealed elevated positivity for MΦs (F4/80) in mouse model ovaries compared with the controls, indicative of increased infiltration of MΦs into the ovarian tissue (*P* = 0.0005; **Figures 6A, B**). In addition, there were distinct expressions and distributions of CD11c^+^ M1-like and CD206^+^ M2-like MΦs between chemotherapy-treated and control ovaries. That is, in comparison to the controls, CD11c^+^ M1-like MΦs were mainly localized surrounding atretic follicles and the corpus luteum in chemotherapy-treated ovaries, indicating that accumulated CD11c^+^ M1-like MΦs might compromise follicular growth (**Figure 7**). By contrast, CD206^+^ M2-like MΦs were primarily distributed in ovarian stroma following chemotherapy, while in the normal ovary, they were predominantly located around growing follicles (**Figure 8**). Given the critical role of M2-like MΦs in tissue fibrosis, we then examined ovarian fibrosis by Masson-stain after mice exposure to CTX/BUS (**Figure 9A**). As shown in **Figure 9B**, CTX/BUS-treated mice exhibited an obvious increase in the positive area of fibrosis within ovarian stroma relative to the controls (*P* = 0.0003). Furthermore, we performed immunofluorescence co-staining of the M2-like MΦs’ marker (CD206) with the fibrotic marker (α-SMA) and found a close localization of CD206 and α-SMA in the ovarian stroma of chemotherapy-treated mice (**Figure 9C**). Then, we used Pearson analysis as an exploratory tool to examine the relationship between fibrosis area, CD206^+^ M2-like MΦs density and the levels of key pro-inflammatory cytokines (IL-1β, IL-6 and TNF-α) within ovaries obtained from the IHC results. Unexpectedly, we observed that within the POI group, there was no significant correlation among fibrosis area, CD206^+^ M2-like MΦs density and the levels of key pro-inflammatory cytokines (IL-1β, IL-6 and TNF-α) (**Supplementary Table 4**). As Pearson analysis only explores association instead of causation, future functional studies are still necessary to determine the relationship between CD206^+^ M2-like MΦs and stromal fibrosis within chemotherapy-induced POI ovaries. Taken together, dynamic alterations in ovarian local MΦs might be involved in the POI progression after chemotherapy.

**Figure 6:**
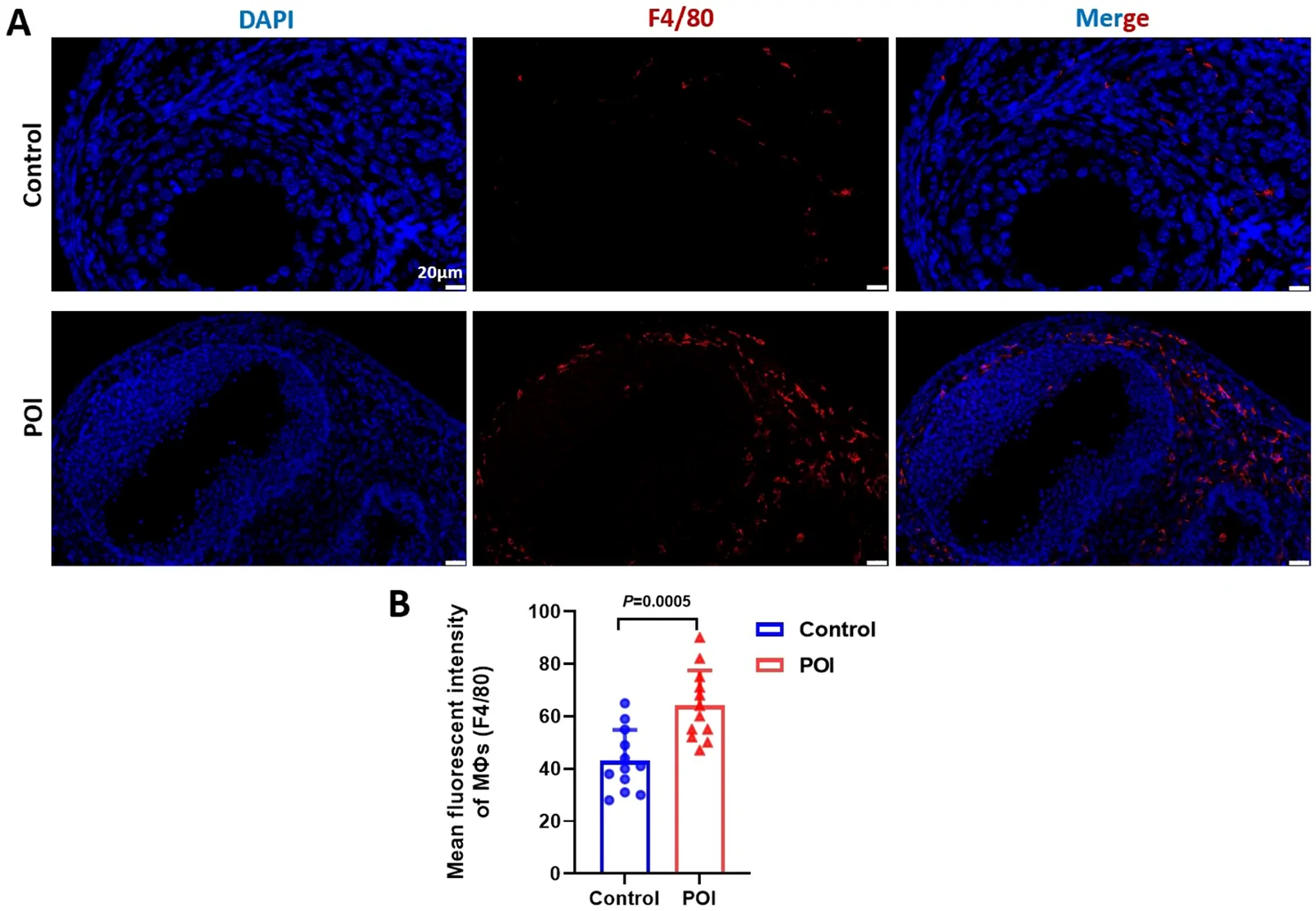
Immunofluorescence analysis of total macrophage distribution in ovaries obtained from control and CTX/BUS-treated mice. **(A)** Representative immunostaining images (×400 magnification) of total ovarian macrophages labelled with F4/80 in each group. Red: F4/80, blue: DAPI. Scale bar: 20μm. **(B)** Fluorescent intensity of F4/80 in ovaries of each group. n=12 mice per group, and 3 independent experiments were performed. Data are represented as the mean ± SD. To avoid the bias, the investigators were blinded to the group assignments of the samples. An unpaired Student’s *t*-test was performed.

**Figure 7:**
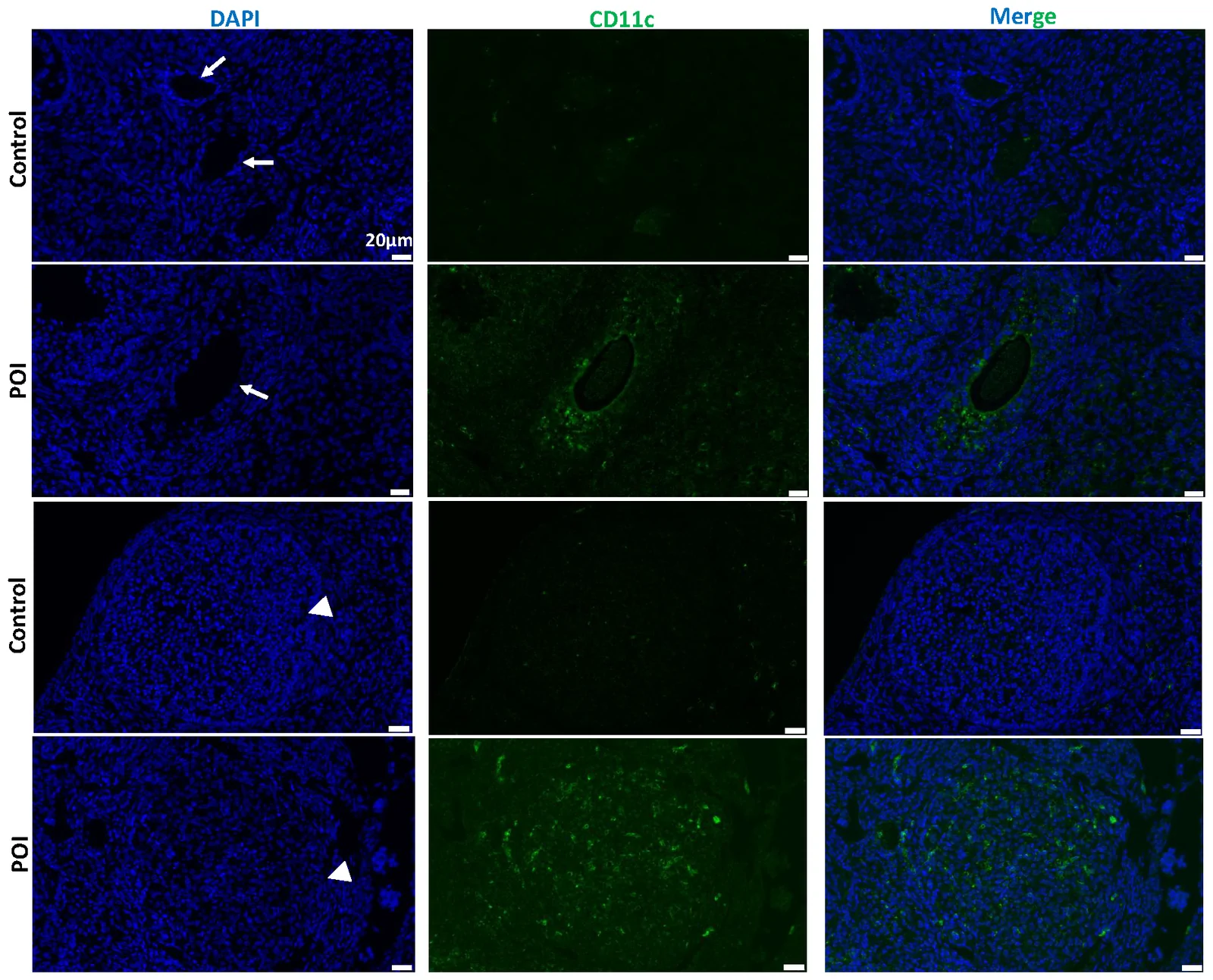
Immunofluorescence analysis of M1-like macrophage distribution in ovaries obtained from control and CTX/BUS-treated mice (×400 magnification). M1-like MΦs were labelled with CD11c in each group. To avoid the bias, the investigators were blinded to the group assignments of the samples. In comparison to the control, CD11c^+^ M1-like MΦs mainly aggregated around atretic follicles (arrow) and corpus luteum (arrowhead) within CTX/BUS-treated ovaries. n=8 mice per group, and 3 independent experiments were performed. Green: CD11c, blue: DAPI. Scale bar: 20μm.

**Figure 8:**
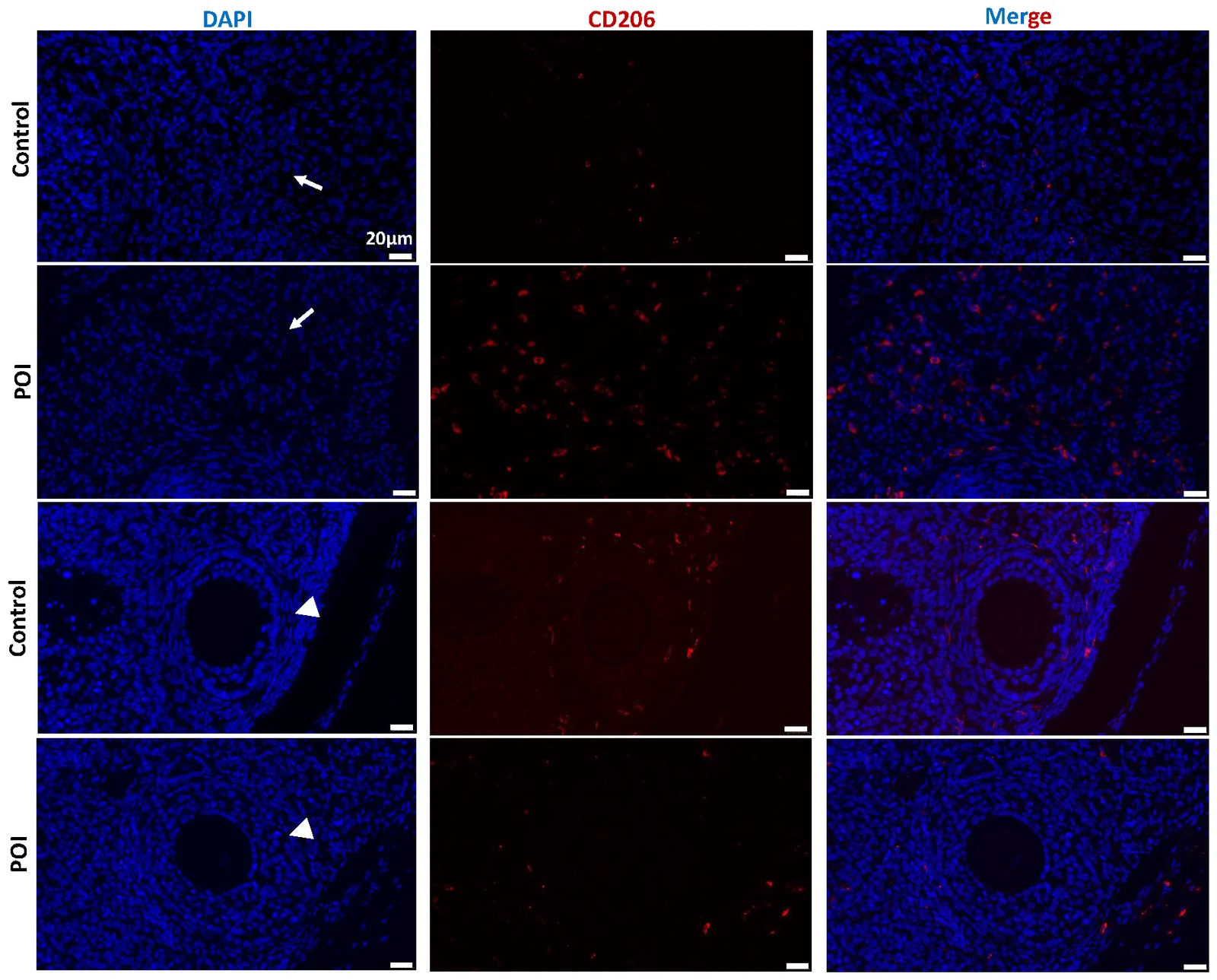
Immunofluorescence analysis of M2-like macrophage distribution in ovaries obtained from control and CTX/BUS-treated mice (×400 magnification). M2-like MΦs were labelled with CD206 in each group. To avoid the bias, the investigators were blinded to the group assignments of the samples. CD206^+^ M2-like MΦs were primarily distributed in the stroma (arrow) of CTX/BUS-treated ovaries, while in control ovaries, they were predominantly located around growing follicles (arrowhead). n=8 mice per group, and 3 independent experiments were performed. Red: CD206, blue: DAPI. Scale bar: 20μm.

**Figure 9:**
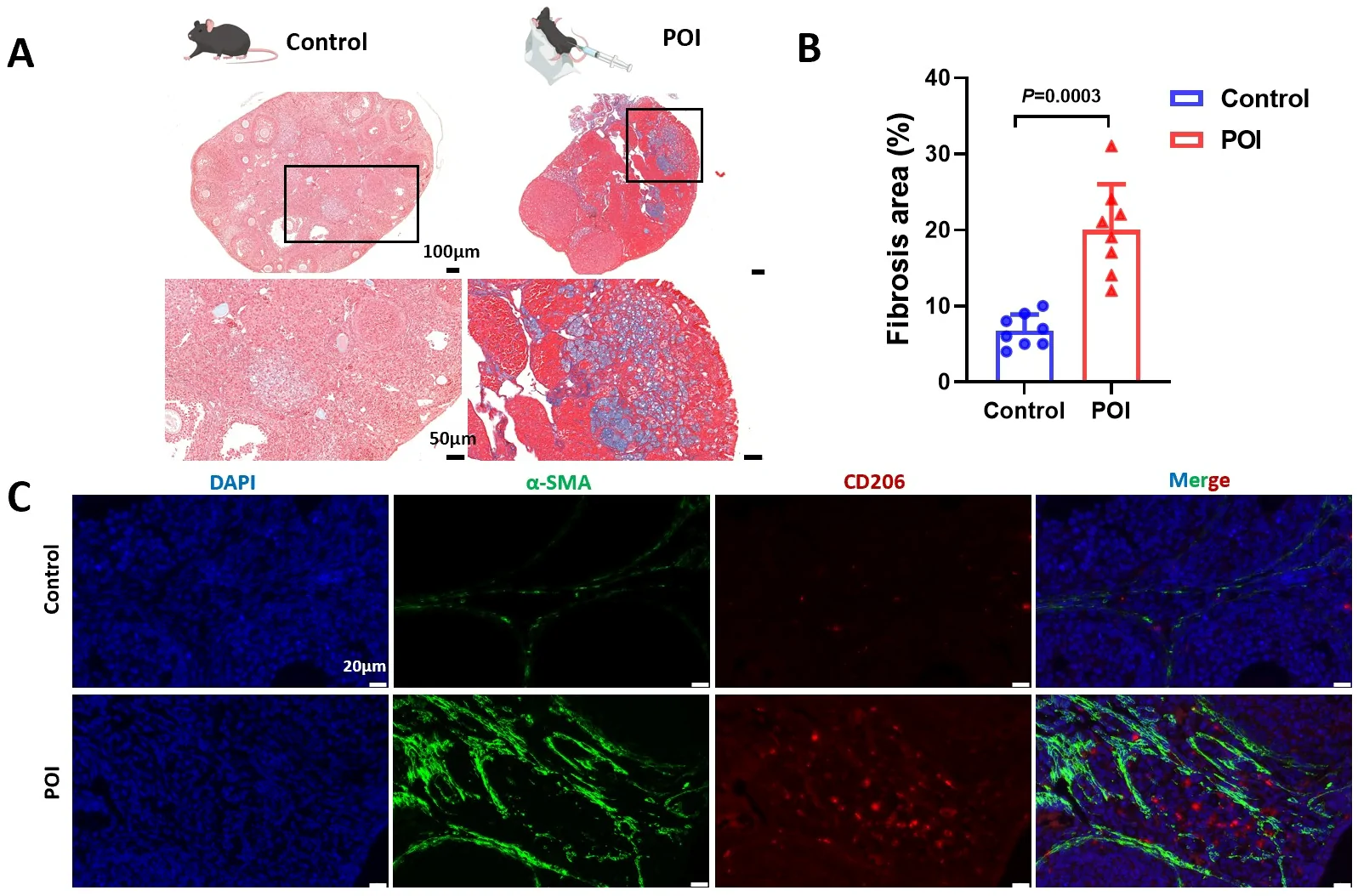
Masson staining analysis of the fibrosis in control and CTX/BUS-treated ovaries. **(A)** Representative images of Masson-stained ovarian tissue sections of each group. Fibrosis areas were indicated by blue color. The top panels (×100 magnification) and below panels (×400 magnification) represent the magnified image of black frames. Scale bar: 100μm (top) and 50μm (below). **(B)** The quantification of ovarian fibrosis by Masson-stain in each group. n=8 mice per group, and 3 independent experiments were performed. Data are represented as the mean ± SD. An unpaired *t*-test with Welch’s correction was performed. **(C)** Representative immunofluorescence staining (×400 magnification) of close localization of α-SMA with CD206 in the ovaries of each group. n=8 mice per group, and 3 independent experiments were performed. Blue: DAPI, green: α-SMA, red: CD206. Scale bar: 20μm. To avoid the bias, the investigators were blinded to the group assignments of the samples.

## Discussion

In this study, we first established a POI mouse model by chemotherapeutic drugs and showed that ovarian function and follicular reserve progressively declined along with increased concentrations of CTX and BUS in mice. Next, using the mouse model, our data suggested an exacerbated inflammation in both the systemic and local ovarian tissues during chemotherapy-induced POI. Finally, flow cytometry analysis revealed an increased polarization of CD11c^+^ M1-like MΦs along with POI development. In addition, using immunofluorescence, we found the differential distribution of M1-like and M2-like MΦs after chemotherapy, with the aggregation of CD11c^+^ M1-like MΦs around atretic follicles and the corpus luteum. By contrast, CD206^+^ M2-like MΦs accumulated around the ovarian stroma, accompanied by excessive fibrosis.

The traditional alkylating agents like CTX and BUS are often utilized to develop mouse POI models via IP injections (42, 43). CTX compromises ovarian function through inducing granulosa cell/oocyte apoptosis and follicle atresia, while BUS accelerates follicular depletion by forming DNA cross-links (26, 44). The combinations of CTX and BUS could produce synergistic effects of ovarian destruction, which produces a stable and long-lasting POI state (at least three weeks) in mice (26, 43, 45). Regarding this combined regimen, there was a conspicuous variability in the dosing protocols of CTX and BUS, including single versus fractionated doses, for inducing mouse POI models (45, 46). For instance, for a single dose IP injection, the CTX doses were reported to range from 80 mg/kg to 200 mg/kg, while the BUS doses were reported to range from 12 mg/kg to 30 mg/kg (26–29). However, to date, the optimal doses of CTX and BUS remain controversial (4, 26). According to the reported drug doses in previous studies, we set and tested a narrow range of doses centered around 90–150 mg/kg CTX combined with 15–30 mg/kg BUS to induce POI in mice. In line with previous studies, our data reveal that chemotherapeutic drugs like CTX and BUS cause ovarian dysfunction in mice, reflected by sex hormone level disruption with increased FSH whereas decreased AMH and E_2_. Moreover, CTX and BUS also cause an increase in damage to ovarian reserve, characterized by a remarkable decline in the number of growing follicles involving primordial, primary, secondary and antral follicles, as well as oocytes retrieved. By contrast, the number of atretic follicles was dramatically increased following chemotherapeutic drug exposure. These data support that CTX and BUS can successfully induce POI in mice. Nevertheless, given that there is currently no consensus on the standardized protocol for using chemotherapeutic drugs to establish a mouse POI model (4, 26, 47), our study design focusing on only a narrow range of CTX and BUS doses is not enough to select the true optimal doses of CTX and BUS. To clarify this issue, further well-designed research, like dose-response studies, longitudinal cohort studies and multivariate parallel controlled studies, is needed.

It is known that the ovarian immunological microenvironment is crucial to support multiple aspects of ovarian physiology, including folliculogenesis, ovulation and corpus luteum formation (4, 15, 17). Our study, using the chemotherapy-induced POI mouse model, showed that the intra-ovarian levels of pro-inflammatory cytokines, including *Il1b*, *Il6* and *Tnfa*, were elevated following CTX and BUS treatment. However, there was no significant change in the expression of anti-inflammatory cytokines *Il4*, *Il10* and *Il13* in ovaries along with the POI development. Consistently, previous studies have revealed rising levels of pro-inflammatory cytokines IL-1β, IL-6, IL-8 and TNF-α, along with declined levels of the anti-inflammatory factors IL-10 and TNF-stimulated gene-6, in mouse or rat ovaries that received CTX/BUS/cisplatin (CIS) chemotherapy (12, 48–50). However, another study showed contrasting findings, which revealed that CTX and BUS treatment reduced the expression of IL-2 and TNF-α, while enhancing the expression of IL-4 in murine ovaries (51). This discrepancy might be partially attributed to dosage-dependent immunomodulatory biphasic effects of alkylating agents. Moderate-to-high single doses of CTX/BUS (e.g., our 150 mg/kg CTX and 15 mg/kg BUS) likely induce intra-ovarian pro-inflammatory cytokine release (IL-1β, IL-6 and TNF-α), potentially through damage-associated molecular patterns (DAMPs) and nucleotide-binding domain and leucine-rich repeat containing family, pyrin domain containing 3 (NLRP3) inflammasome activation (52, 53). By contrast, higher single drug doses (e.g., 200 mg/kg CTX or 30 mg/kg BUS) or fractionated regimens may exert immunosuppression effects on ovarian immunity by eliciting compensatory anti-inflammatory responses with increased IL-4 expressions, whereas TNF-α and IL-2 expressions decrease (4, 51, 54, 55). While the mechanism underlying this phenomenon remains unclear, it is reportedly associated with the disruption of the Th1/Th2 balance in ovaries caused by chemotherapy (4, 54). Apart from dosage factors, these inconsistent cytokine results across studies are possibly due to the variability in sampling time points and detection methods (4). Future time-course and dose-response studies are warranted to disentangle primary immune dysregulation from secondary compensatory responses in chemotherapy-induced POI.

In addition, our study further investigated the systemic levels of pro-inflammatory cytokines IL-6, IL-18 and TNF-α. We found that the serum levels of IL-6 and IL-18 were dramatically increased, whereas there was no significant change in the serum TNF-α levels following CTX/BUS treatment in mice. These findings are partially consistent with prior studies suggesting increased serum concentrations of IL-6 and TNF-α in rat models following CTX treatment (56). In addition to the analysis in rodent animals, another study further revealed elevated levels of serum IL-6 and monocyte chemoattractant protein-1 (MCP-1) in both humans and rhesus macaques after chemotherapy (57). The chemokine MCP-1 was involved in attracting substantial innate immune cells like monocytes and MΦs to the ovary (57). Nevertheless, our current work lacks the experiments to clarify the mechanistic association between the systemic-local inflammation and ovarian MΦs changes or ovarian injury. It has been reported that the chemotherapy brought an initial ovarian toxic hit resulting in accumulated damaged cells (debris), which triggers DAMPs, senescence-associated secretory phenotypes (SASP), and NLRP3, without sparking a compensatory anti-inflammatory response (52, 53, 57–59). They in turn recruit monocytes and MΦs into the ovary and induce systemic-local secondary inflammations leading to excessive follicle apoptosis and atresia (17, 57, 59). Under the inflammatory microenvironment within the ovaries, the MΦs might polarize heavily toward an M1-like (pro-inflammatory) phenotype instead of an M2-like (anti-inflammatory) phenotype (17). The M1-like MΦs produce multiple pro-inflammatory cytokines including TNF-α, IL-1α/β, IL-6, IL-12, IL-18 and IL-23, and this ultimately develops into a self-perpetuating vicious cycle, further exacerbating follicle depletion and ovarian injury (17, 52, 60). Collectively, our data suggest an elevation of inflammatory status in both peripheral blood and ovarian tissues during chemotherapy, which may be associated with ovarian functional decay. However, this conclusion should be interpreted with caution until mechanistic or functional data become available.

Emerging studies have revealed that MΦs constitute an essential component of the ovarian immune microenvironment with binary polarization status of M1/M2-like MΦs (4, 22, 61, 62). Our flow cytometry analysis of ovarian MΦs using canonical surface markers, CD11c for M1 and CD206 for M2, indicated that in comparison to controls, CTX/BUS-treated mice had a conspicuous increase in the proportion of CD11c^+^ M1-like MΦs, while no significant difference was observed in the proportion of CD206^+^ M2-like MΦs within ovaries. Meanwhile, using immunofluorescence, our work showed extensive MΦs infiltration in ovaries of CTX/BUS-treated mice, relative to those of the controls. Additionally, the immunostaining results revealed differential distribution of M1-like and M2-like MΦs in mouse ovaries treated with CTX/BUS. Specifically, we found the accumulation of CD11c^+^ M1-like MΦs around atretic follicles and corpus luteum, whereas CD206^+^ M2-like MΦs were predominantly located in ovarian stroma along with CTX/BUS-induced POI. Our results are in line with previous mouse studies, indicating that numerous MΦs infiltrated the ovary after CTX treatment (13). Consistently, it has been revealed that the M1-like MΦs polarization is increased in response to the inflammatory tissue environment (17, 62). Previous studies have suggested that after chemotherapy, disruption of M1/M2-like MΦs’ balance and elevated M1-like MΦs’ activities directly or indirectly impede the development of ovarian granulosa cells and steroid hormone synthesis, ultimately resulting in follicular degeneration in ovaries (15). Therefore, it can be inferred that chemotherapy may be associated with M1-like MΦs polarization and enhanced inflammation, potentially contributing to ovarian damage. Nevertheless, this hypothesis requires in-depth mechanistic research and MΦs intervention assays for validation.

By contrast, the M2-like MΦs are stimulated by anti-inflammatory factors, including IL-4, IL-10 and IL-13 (17, 19). These can partially explain our results showing increased CD11c^+^ M1-like MΦs, whereas CD206^+^ M2-like MΦs are unchanged in the chemotherapy-treated ovaries. Moreover, the M2-like MΦs synthesize IL-10, transforming growth factor beta (TGF-β), fibroblast growth factor (FGF) and platelet-derived growth factor (PDGF), which are associated with the resolution of inflammation, tissue repair and fibrosis (17, 63, 64). Nonetheless, our Pearson analysis has shown that CD206^+^ M2-like MΦs accumulation in the stroma is not associated with excessive fibrosis in CTX/BUS-treated ovaries. This discrepancy may be due to small sample sizes, limited statistical power, or biological heterogeneity among animals. Considering that Pearson analysis cannot distinguish causality or the co-variation is incidental, future mechanistic studies are required to determine whether a direct relationship exists between the CD206^+^ M2-like MΦs and ovarian fibrosis during chemotherapy.

It is undeniable that our study has several limitations. This work lacks the evidence to show whether inflammation and MΦs changes precede or follow follicle loss during chemotherapy. In future research, a time-course analysis after chemotherapy to determine whether ovarian immune changes are a primary driver or secondary effects of follicle loss is highly warranted. Moreover, although CD11c and CD206 remain accepted markers for distinguishing MΦs subtypes in mouse ovaries, it is worth noting that using only these markers to define M1/M2-like polarization oversimplifies MΦs heterogeneity in our study (20, 21). That is because MΦs phenotypes exist along a functional continuum rather than as rigid binary states in the ovarian microenvironment (17). More additional M1/M2 markers (e.g., iNOS and CD86 for M1 and CD163 for M2) are therefore required to precisely delineate the dynamics of ovarian MΦs polarization during chemotherapy-induced POI. In addition to an in-depth exploration of MΦs’ functions, future studies are still needed to examine a causal relationship between MΦs’ polarizations, inflammation and follicle depletion through MΦs intervention or polarization modulation experiments in chemotherapy-induced POI. This will provide more mechanistic and therapeutic insights into POI caused by chemotherapy.

## Conclusions

Our preliminary study indicates the intensive inflammation that occurs in mouse ovaries during chemotherapy-induced POI. This inflammation might be linked to a disruption in the M1/M2-like MΦs’ ratio, which could potentially contribute to POI development. Nonetheless, this finding should be interpreted cautiously until additional mechanistic or functional data is revealed. Future interventional studies are required to determine whether targeting the ovarian microenvironment and MΦs polarization would ameliorate chemotherapy-induced POI. Indeed, due to the lack of published literature on the field of MΦs polarization and chemotherapy-induced POI, it is rather difficult to compare our data with those of others. Future studies are still needed to elucidate the mechanisms that are responsible for altered dynamics of MΦs in ovaries following chemotherapy.

## Data Availability

The original contributions presented in the study are included in the article/**Supplementary Material**. Further inquiries can be directed to the corresponding author/s.
