# Supplementary material for: Evaluation of inflammatory status and macrophage polarization in premature ovarian insufficiency mice induced by chemotherapy
Source: Front Endocrinol (Lausanne). 2026 Oct 12;17:1798400. doi: 10.3389/fendo.2026.1798400 (PMC13696237; doi:10.3389/fendo.2026.1798400)
Supplement: Supplementary file 1 [file Table1.docx]

**Supplementary Table 1** The initial body weights (g) of all the mice.

|  | **Control** | **Group I** | **Group II** | **Group III** | **Group IV** |
| --- | --- | --- | --- | --- | --- |
|  | 23.1 | 19.2 | 22.7 | 20.0 | 20.5 |
|  | 22.7 | 22.9 | 23.0 | 22.7 | 19.0 |
|  | 20.4 | 23.3 | 19.6 | 22.4 | 20.9 |
|  | 20.6 | 22 | 22.9 | 19.4 | 21.0 |
|  | 21.4 | 19.5 | 20.6 | 20.9 | 22.6 |
|  | 19.8 | 21.1 | 19.7 | 21.9 | 23.1 |
|  | 19.2 | 21.0 | 21.8 | 22.0 | 19.8 |
|  | 22.5 | 21.9 | 22.6 | 21.1 | 22.2 |
| Mean ± SD | 21.21±1.442 | 21.36±1.472 | 21.61±1.440 | 21.30±1.166 | 21.14±1.412 |

Data are represented as the mean ± SD. Among the groups, an ANOVA with Tukey’s Multiple Comparison Test was performed. Control vs. Group I, *P*=0.9995; Control vs. Group II, *P*= 0.9779; Control vs. Group III, *P*>0.9999; Control vs. Group IV, *P*>0.9999; Group I vs. Group II, *P*=0.9963; Group I vs. Group III, *P*>0.9999; Group I vs. Group IV, *P*= 0.9975; Group II vs. Group III, *P*=0.9912; Group II vs. Group IV, *P*=0.9588; Group III vs. Group IV, *P*=0.9993.
