# Supplementary material for: Evaluation of inflammatory status and macrophage polarization in premature ovarian insufficiency mice induced by chemotherapy
Source: Front Endocrinol (Lausanne). 2026 Oct 12;17:1798400. doi: 10.3389/fendo.2026.1798400 (PMC13696237; doi:10.3389/fendo.2026.1798400)
Supplement: Supplementary file 2 [file Table2.docx]

**Supplementary Table 2** The antibodies for immunofluorescence.

| **Antibodies** | **Name** | **Cat#** | **Dilutions** | **Manufacturer** |
| --- | --- | --- | --- | --- |
| The primary antibodies | Rabbit anti-F4/80 | GB113373 | 1:500 | Servicebio |
|  | Rabbit anti-CD11c | GB11059 | 1:400 | Servicebio |
|  | Rabbit anti-CD206 | GB113497 | 1:400 | Servicebio |
|  | Monoclonal mouse anti-alpha smooth muscle actin (anti-α-SMA) | GB121364 | 1:1000 | Servicebio |
| The secondary antibodies | Cy3-conjugated goat anti-rabbit IgG for F4/80 staining | GB21303 | 1:100 | Servicebio |
|  | HRP-conjugated goat anti-rabbit IgG and iF488-Tyramide for CD11c staining | GB23303  G1231 | 1:500  1:500 | Servicebio |
|  | HRP-conjugated goat anti-rabbit IgG and CY3-Tyramide for CD206 staining | GB23303  G1223 | 1:500  1:500 | Servicebio |
|  | Alexa Fluor® 488-conjugated goat anti-mouse IgG (H+L) for α-SMA staining | GB25301 | 1:400 | Servicebio |
