# Supplementary material for: Evaluation of inflammatory status and macrophage polarization in premature ovarian insufficiency mice induced by chemotherapy
Source: Front Endocrinol (Lausanne). 2026 Oct 12;17:1798400. doi: 10.3389/fendo.2026.1798400 (PMC13696237; doi:10.3389/fendo.2026.1798400)
Supplement: Supplementary file 3 [file Table3.docx]

**Supplementary Table 3** The antibodies for immunohistochemistry.

| **Antibodies** | **Name** | **Cat#** | **Dilutions** | **Manufacturer** |
| --- | --- | --- | --- | --- |
| The primary antibodies | Rabbit polyclonal antibody to IL-1α | DF6893 | 1:50 | Affinity |
|  | Rabbit polyclonal antibody to IL-1β | GB11113 | 1:300 | Servicebio |
|  | Rabbit polyclonal antibody to IL-6 | GB11117 | 1:100 | Servicebio |
|  | Rabbit polyclonal antibody to TNF-α | GB115702 | 1:300 | Servicebio |
| The secondary antibodies | S-Vision Poly-HRP-conjugated goat anti-rabbit IgG (H+L) | G1302 | Ready-to-use without dilution | Servicebio |
