# Supplementary material for: Evaluation of inflammatory status and macrophage polarization in premature ovarian insufficiency mice induced by chemotherapy
Source: Front Endocrinol (Lausanne). 2026 Oct 12;17:1798400. doi: 10.3389/fendo.2026.1798400 (PMC13696237; doi:10.3389/fendo.2026.1798400)
Supplement: Supplementary file 4 [file Table4.docx]

**Supplementary Table 4** Pearson correlation analysis separately within the control group and within the POI group.

| **Groups** | **Correlation** | ***r* values** | ***P-values*** |
| --- | --- | --- | --- |
| Within the control group (n=8) | Number of CD206^+^ M2-like MΦs/HPF & Fibrosis area (%) | 0.8335 | 0.0101 |
|  | Number of CD206^+^ M2-like MΦs/HPF & IL-1β level (average optical density) | 0.0504 | 0.9056 |
|  | Number of CD206^+^ M2-like MΦs/HPF & IL-6 level (average optical density) | -0.5115 | 0.1951 |
|  | Number of CD206^+^ M2-like MΦs/HPF & TNF-α level (average optical density) | 0.0508 | 0.9049 |
|  | Fibrosis area (%) & IL-1β level (average optical density) | 0.0602 | 0.8873 |
|  | Fibrosis area (%) & IL-6 level (average optical density) | -0.5717 | 0.1388 |
|  | Fibrosis area (%) & TNF-α level (average optical density) | -0.0571 | 0.8931 |
| Within the POI group (n=8) | Number of CD206^+^ M2-like MΦs/HPF & Fibrosis area (%) | -0.4433 | 0.2712 |
|  | Number of CD206^+^ M2-like MΦs/HPF & IL-1β level (average optical density) | -0.3688 | 0.3687 |
|  | Number of CD206^+^ M2-like MΦs/HPF & IL-6 level (average optical density) | 0.3049 | 0.4627 |
|  | Number of CD206^+^ M2-like MΦs/HPF & TNF-α level (average optical density) | 0.3608 | 0.3800 |
|  | Fibrosis area (%) & IL-1β level (average optical density) | 0.4296 | 0.2881 |
|  | Fibrosis area (%) & IL-6 level (average optical density) | 0.3027 | 0.4661 |
|  | Fibrosis area (%) & TNF-α level (average optical density) | -0.6894 | 0.0586 |

n=8 mice, denoting the total number of biologically independent animals included in the correlation analysis, and 3 independent experiments were performed.
